# RETRACTION: MCOLN1 Promotes Proliferation and Predicts Poor Survival of Patients with Pancreatic Ductal Adenocarcinoma

**DOI:** 10.1155/dim/9850902

**Published:** 2025-05-06

**Authors:** Disease Markers

RETRACTION: Z.-D. Hu, J. Yan, K.-Y. Cao, Z.-Q. Yin, W.-W. Xin, and M.-F. Zhang. “MCOLN1 Promotes Proliferation and Predicts Poor Survival of Patients with Pancreatic Ductal Adenocarcinoma,” *Disease Markers*, no. 2019 (2019): 1–9. https://doi.org/10.1155/2019/9436047.

The above article, published online on 5 August 2019 in Wiley Online Library (wileyonlinelibrary.com), has been retracted by John Wiley & Sons Ltd.

The retraction has been agreed following an investigation of the concerns raised by *Hoya camphorifolia* on PubPeer [1], which identified multiple instances of inappropriately overlapping figures.

Specifically:

  - Figure 2c: The immunoblot assay bands corresponding to Ki67 and *β*-actin expression in the Bx-PC3 cells have been duplicated in Figure 2b of [2] to represent KIF18A and *β*-actin expression in the H1975 cells.
  - Figure 3a: The first, third, and fourth tumors in the top row appear identical to the first, fourth, and fifth tumors in the bottom row of Figure 5a in [3].
  - Figure 3b: The Western blot is a mirror image of Figure 6c from [4], while denoting different proteins.

As a result of the investigation, the data and conclusions of this article are considered unreliable.

The authors were informed of the decision to retract the article but did not provide a response.
